# Multidirectional Piezoelectric Vibration Energy Harvester Based on Cam Rotor Mechanism

**DOI:** 10.3390/mi14061159

**Published:** 2023-05-30

**Authors:** Xin Jiang, Yan Liu, Jiaming Wei, Haotian Yang, Bin Yin, Hongbo Qin, Weidong Wang

**Affiliations:** 1 School of Mechano-Electronic Engineering, Xidian University, Xi’an 710071, China; 21041212042@stu.xidian.edu.cn (X.J.); jmwei@stu.xidian.edu.cn (J.W.); 17040210008@stu.xidian.edu.cn (H.Y.); 20041211945@stu.xidian.edu.cn (B.Y.); qhb0920qhb@xidian.edu.cn (H.Q.); 2 CityU-Xidian Joint Laboratory of Micro/Nano Manufacturing, Shenzhen 518057, China

**Keywords:** piezoelectric vibration energy harvester, multidirectional, cam rotor, wireless sensor network

## Abstract

The techniques that harvest mechanical energy from low-frequency, multidirectional environmental vibrations have been considered a promising strategy to implement a sustainable power source for wireless sensor networks and the Internet of Things. However, the obvious inconsistency in the output voltage and operating frequency among different directions may bring a hindrance to energy management. To address this issue, this paper reports a cam-rotor-based approach for a multidirectional piezoelectric vibration energy harvester. The cam rotor can transform vertical excitation into a reciprocating circular motion, producing a dynamic centrifugal acceleration to excite the piezoelectric beam. The same beam group is utilized when harvesting vertical and horizontal vibrations. Therefore, the proposed harvester reveals similar characterization in its resonant frequency and output voltage at different working directions. The structure design and modeling, device prototyping and experimental validation are conducted. The results show that the proposed harvester can produce a peak voltage of up to 42.4 V under a 0.2 g acceleration with a favorable power of 0.52 mW, and the resonant frequency for each operating direction is stable at around 3.7 Hz. Practical applications in lighting up LEDs and powering a WSN system demonstrate the promising potential of the proposed approach in capturing energy from ambient vibrations to construct self-powered engineering systems for structural health monitoring, environmental measuring, etc.

## 1. Introduction

In recent years, we have witnessed great challenges in the global environment that are induced by the wide usage of carbon energy. Pursuing progress in clean, renewable and sustainable energy has become a critical issue to replace conventional energy sources [1]. In the meantime, the widespread application of high-performance devices in wireless sensor networks (WSNs) and the Internet of Things (IoT), e.g., wearable electronics, sensors, tags, data transmitters and microprocessors, has extended their appearance in building monitoring, climate information acquisition, smart factories/traffic/homes, personal healthcare and environmental monitoring, which is greatly excited by advances in material science, manufacturing techniques and ecofriendly concepts [2,3,4,5,6,7]. However, the power supply for WSNs/IoT is still dominated by conventional electrochemical batteries, whose periodic replacement inevitably brings great environmental problems and maintenance challenges, whereas the low-power feature of these devices also provides great feasibility in taking advantage of ambient mechanical energy to sustainably power these systems. Therefore, a small-scale mechanical energy harvesting technique, which generates electricity via capturing renewable but commonly dissipated environmental energy, has attracted increasing attention in recent years [8,9].

Generally, mechanical energy in the daily environment mainly holes up in low-frequency (often <5 Hz), unoriented vibrations, rotations and flows [10,11]. This feature requires energy harvesters, whether piezoelectric [12,13], triboelectric [14], or electromagnetic [15], with a desirable frequency response and multidirectional capacity, to fully adapt themselves to daily mechanical excitations. Particularly, for the vibrations in wind-induced motions [16], building shakings [17], water waves [18], and motor running [19], harvesting efficiency greatly depends on the structural resonance and shows obvious deviations among different frequency ranges and directions. When the resonant frequency of the harvester cannot cover the vibration frequency, it will become difficult to efficiently transmit mechanical energy to a conversion transducer. Similarly, inconsistency between the vibration direction and harvester modal shape also influences conversion efficiency.

Harvesters, based on different principles, possess varied adaptabilities. A triboelectric generator has a superior performance in harvesting energy from low-frequency and irregular motion, but the lack of a modulation mechanism for transforming 3D vibration to one-way motion will limit the output power when it faces multidirectional excitation [20]. Similarly, an electromagnetic harvester generates electricity based on the relative motion between the magnetic field and the coil. The modulation mechanism also dominates the harvesting performance [21]. With the absence of high-performance modulators, the above two schemes may need to assemble multiple units together to deal with the multidirectional circumstance, which will cause problems in the device size, weight and cost. A piezoelectric harvester responds to external vibration by structural bending, and the strain energy is directly transmitted to the bonded piezoelectric transducer. Although a piezoelectric device cannot accomplish a power density as good as an electromagnetic one, it performs well when dealing with multidirectional cases because it is unnecessary to strictly define the relative movement between the different elements.

A piezoelectric vibratory energy harvester (PVEH) based on a cantilevered structure can easily cover the frequency spectrum of daily vibrations but cannot well fulfill the multidirectional task. Researchers have effectively increased its working direction by setting up multiple independent beams and introducing additional components. Deng et al. proposed a double-branched structure with three cantilever beams to achieve a bidirectional PVEH with four natural frequencies below 22 Hz. Due to its multimodal and bidirectional capacity, the obtained average power density is over 3800 times the compared single cantilever scheme [22]. Wang et al. utilized spring buffering and magnetic coupling effects to capture three-dimensional vibrations and transform them to the metal shim and inner beam [23]. As for single-beam approaches, cantilever–pendulum and twist-beam structures have proved their capacity to harvest multidirectional vibrations [24,25,26]. More recently, several remarkable configurations have been put forward to extend the operating scope of a single-beam scheme. Firstly, mounting the cantilever onto a self-adaptive direction regulator is a practical method to endow all-around directional sensitivity for the harvester. Inspired by the vibrational stabilization of hummingbirds and mosquitos in hovering flight, the cantilever was clamped using a coaxial rotatable fixture to realize a superior vibrational stability under multidimensional vibrations. Compared with a conventional cantilever scheme, the proposed harvesters could effectively harvest energy from vibrations in any direction [27,28]. Then, the cantilever shape can be modified to simultaneously sense bending and torque loads. It has been proved that a quarter-circular arc piezoelectric beam can harvest vibration energy in three directions [29]. Based on the conventional cantilever–pendulum approach, Qin’s group used liquid as the energy-capturing medium to simultaneously achieve ultralow frequency, low intensity and multidirectional energy harvesting [30,31]. The liquid was stirred using horizontal or vertical vibrations, and the induced waves were sensed by the floater–lever array to bend the piezoelectric beam. Sheng et al. hung a pendulum between two vertical beams, and the ability of the sphere-shaped mass to capture arbitrarily the directional vibration provided the harvester with an operation bandwidth of 3.6 Hz, 2.1 Hz and 3.6 Hz in three directions [32]. Simultaneously, several improved beam structures with inherent multidirectional capacity have been introduced into the construction of PVEHs. For example, a U-shaped beam can harvest energy from bidirectional vibrations [33], and the capacity can be extended to tri-dimension by adding a pendulum [34], introducing a cross-connected configuration [35] or being modified into an M shape [36]. Obviously, most of the referred multidirectional schemes still operate based on the inertia-force-induced bending of beams. The multiple-beam approaches can independently optimize the parameter of each unit to ensure the consistency of the output in different directions, but the occupied space is still a critical issue. In the single-unit approaches, the harvesting of multidirectional vibration relies on different bending states, inevitably bringing about an inconformity in the resonant frequency or output voltage. For example, the twist cantilever in [24] can generate a voltage of 16.48 V at 22.37 Hz under a 0.5 g vertical vibration, but the value turns to 41.47 Hz and 2.77 V when the device harvests a horizontal vibration. Considering these existing issues, there is still a need to further expand the structural scope of multidirectional PVEHs.

This paper, thus, introduces a cam rotor mechanism into the construction of a low-frequency multidirectional PVEH to pursue a similar harvesting performance in different directions. Unlike the conventional way of directly loading inertia force to the piezoelectric beam in every direction, the vertical vibration is first converted into a reciprocating circular motion by the cam rotor, and then a corresponding centrifugal acceleration is loaded to trigger two pairs of orthogonally settled piezoelectric beams. In the meantime, the horizontal vibrations can be directly harvested by the same beams under the conventional bending pattern. The utilization of the same beam group ensures similar dynamical features for the proposed PVEH on both vertical and horizontal occasions. The originality and contributions of this paper are summarized as follows:

(a) The cam rotor mechanism is first utilized to transform the vertical excitations to reciprocating circular motions, which can produce a dynamic centrifugal acceleration to excite piezoelectric beams;
(b) The same beam group is used to realize a similar performance when harvesting multidirectional vibrations.

The design concept and basic mechanical properties of the proposed approach were described with the necessary modeling and simulation. Then, the device prototyping and characterization experiments were conducted. Simple applications of lighting up LEDs and powering wireless sensing nodes were validated to prove the potential of the proposed prototype as a possible substitution for electrochemical batteries in WSNs/IoT, which would be favorable assistance for constructing self-powered monitoring systems in buildings and natural environments.

## 2. Design and Analysis

### 2.1. Design Concept

**Figure 1:**
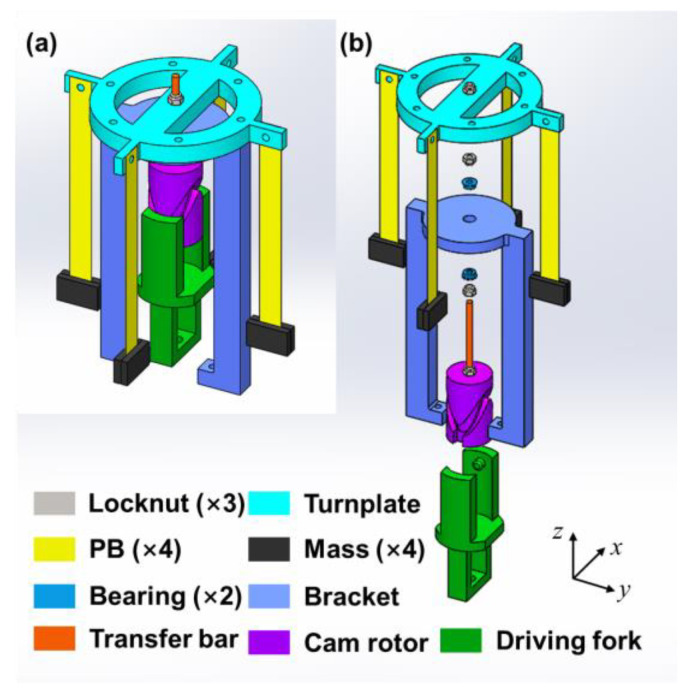
The cam-rotor-based PVEH: (**a**) schematic diagram and (**b**) exploded view.

Figure 1 shows the schematic design and exploded view of proposed PVEH, which mainly consists of two pairs of piezoelectric beams (PBs) with masses at their free ends, a turnplate, a bracket, a cam rotor, a driving fork and a transfer bar. The PBs, which will be deflected by the inertia force from masses, are, respectively, mounted to the four connectors of turnplate. Their length direction is aligned with the z-axis direction. The turnplate is fixed to the transfer bar by thread joint to obtain the circular motion generated by cam rotor. The cam rotor and driving fork constitute the core unit that converts vertical z-axis vibration into a circular motion in x–y plane, whose operating principle will be discussed in the following subsection. The bracket plays the role of fixing PVEH onto the target object and is isolated from the transfer bar by a set of bearings to avoid obstructing the structural rotation. The cam rotor has two spiral grooves symmetrically on its periphery to guarantee the production of desired reciprocating circular motion. In the driving fork, two humps are designed to form a kinematic pair with the grooves. A mounting hole is settled at the bottom of driving fork for connecting the device with vibrated surface. The transfer bar has screw thread on it, cooperating with the locknuts, to assemble all the parts together.

**Figure 2:**
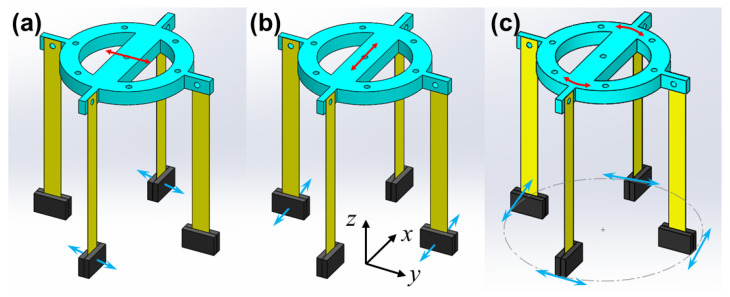
The direction of loaded accelerations on PBs under (**a**) *x*-axis or (**b**) *y*-axis horizontal vibration and (**c**) *z*-axis vertical vibration (Red arrows for vibration motion and cyan ones for acceleration).

The working principle of proposed PVEH can be divided into two cases: the horizontal vibration (HV) circumstance and vertical vibration (VV) circumstance, as shown in Figure 2. The acquisition of HVs (along x- or y-axis) is directly fulfilled by the two pairs of orthogonally located PBs. When HV appears, an inertia force will be loaded to the PBs, whose normal direction is parallel to the acceleration component. The stimulated PB will generate a strain in piezoelectric patch, and then certain electricity is produced according to direct piezoelectric effect. Herein, the piezoelectric patch is made of lead zirconate titanate (PZT) ceramic, and the details of its piezoelectric effect can be found in [10]. As for VVs (along z-axis), the vertical-to-circular motion conversion of cam rotor mechanism plays the key role. Firstly, the driving fork receives displacement from the vibrated surface, and the cam rotor will be driven to perform a reciprocating circular motion. Subsequently, the turnplate achieves the induced circular motion from transfer bar, and a centrifugal acceleration will be loaded to the masses in PBs. With the combination of two horizontal directions from orthogonal PB pairs and an additional vertical direction from cam rotor mechanism, the energy in tri-directional vibrations can be successfully acquired by the PVEH. It is of note that the optimal frequency of proposed PVEH for multidirectional vibrations can be easily uniformized due to the sharing of same PB pairs under different cases.

### 2.2. Analysis of Motion Conversion

**Figure 3:**
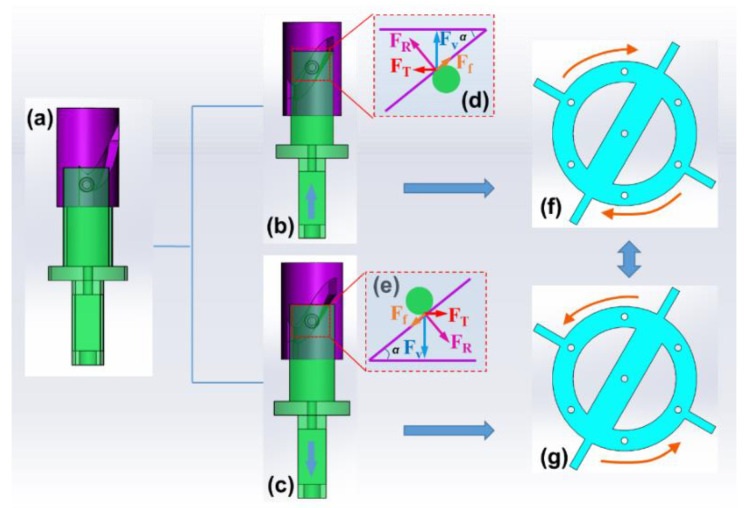
Operating principle of cam rotor for converting vertical displacement into rotation: (**a**) assembly between cam rotor and driving fork; the movement (**b**), slope–block pair (**d**) and rotation of turnplate (**f**) during the upward stage of vertical vibration; the movement (**c**), slope–block pair (**e**) and rotation of turnplate (**g**) during the downward stage vertical vibration.

As mentioned above, the kinematic pair between cam rotor and driving fork play a key role in converting the vertical displacement into a reciprocating circular motion. This subsection mainly focuses on the principle of this motion conversion. Figure 3 illustrates the process of rotor activation and force analysis under the vibration-induced up and down movements of driving fork. When the driving fork is excited by the upward stage of vertical vibration (Figure 3b), the humps will squeeze the upper surface of groove, providing a driving force to rotate the cam rotor. As shown in Figure 3d, the corresponding kinematic pair can be regarded as a slope–block pair, and the block (namely the hump in driving fork) exhibits a movement toward the included angle. The vertically loaded force *F_v_* comes from the vibrated surface, and the reactive force *F_R_* is the support force from slope. Then, a horizontal thrust *F_T_* appears in the contact area, generating a torque to rotate the turnplate clockwise. Similarly, when the driving fork is excited by downward displacement (Figure 3c), the humps will squeeze the lower surface of groove, and the slope–block mode in Figure 3e is also valid. The new thrust *F_T_* exhibits an inverse direction and drives the turnplate to contrarotate. Under the alternating action of thrusts in these two stages, the circular motion of turnplate will reciprocate, applying a dynamic stimulation to PBs.

The relationship between *F_v_* and *F_T_* can be written in Equation (1), where *α* is the included angle of slope, and *μ* is the friction coefficient for the slope–block pair. Assuming *r* is the distance between the center of cam rotor and the loading point of *F_T_*, the driving torque *T* can be expressed in Equation (2):

(1) $$F_{T}=F_{v}\sin\alpha \cos\alpha -\mu F_{v}{cos}^{2}\alpha ,$$

(2) $${T=2rF}_{T}={2rF}_{v}\cos\alpha \left(\sin\alpha -\mu \cos\alpha \right).$$

**Figure 4:**
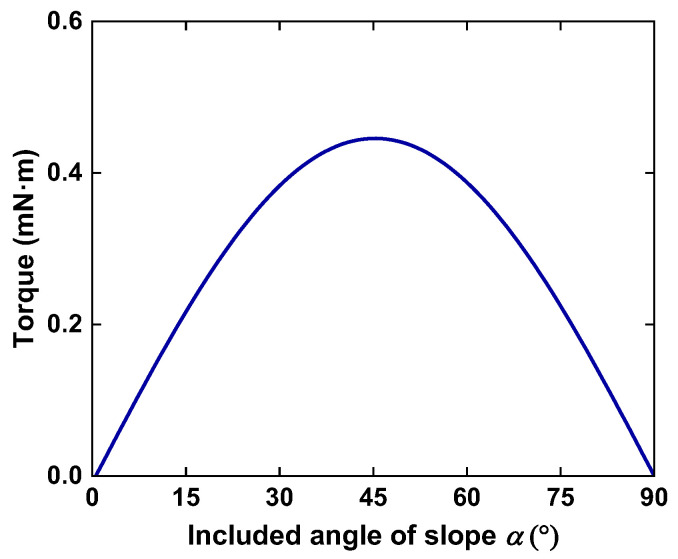
The driving torque on cam rotor under different included angles.

With the aforementioned equations, the driving torque *T* as a function of included angle *α is* shown in Figure 4, in which *F*_*v*_ = 50 mN, *r* = 9 mm and *μ* = 0.01. It can be seen that the generated torque varies with the included angle *α*, and the maximum value appears at *α* = 45°. The direction of torque changes with the direction of vibration, driving the cam rotor and turnplate to make a reciprocating circular motion.

### 2.3. Model for Piezoelectric Beam

**Figure 5:**
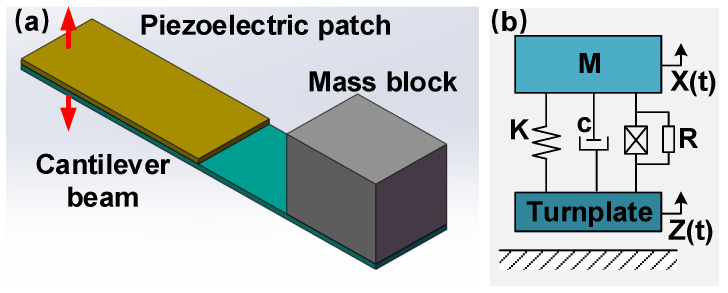
The cantilever piezoelectric energy harvester mode for PBs: (**a**) schematic diagram and (**b**) equivalent lumped-parameter mode.

When triggered by accelerations, no matter the linear acceleration from HV or centrifugal acceleration induced by VV, the PBs will produce certain electrical energy. Generally, the PB can be regarded as a cantilever piezoelectric energy harvester, which consists of a cantilever beam with one end fixed at the turnplate, a mass block attached on the other end and a piezoelectric patch near its fixed end (Figure 5a). If ignoring the mass of cantilever and regarding the mass block as a rigid body, the PB can be considered a 1-DOF mass-spring-damper mechanical system. The system equivalent mass is determined by the weight of mass block, and the stiffness is dominated by the substrate and PZT patch. The electromechanical coupling model of this device can be indicated by the equivalent lumped-parameter dynamic system in Figure 5b. Then, the dynamic equations of this model are written as:

(3) $$M\ddot{X}+c\dot{X}+KX+\theta V=-M\ddot{Z},$$

(4) $$C_{P}\dot{V}+\frac{V}{R}=\theta \dot{X}$$

*M*, *c*, *K* are the equivalent mass, damping and stiffness of this system; *θ* and *C_P_* are the coupling factor and internal capacitance of used piezoelectric patch; *V* is the voltage over the external resistance load *R* under the turnplate displacement *Z*; and *X* is the relative displacement of mass block. Assuming the vibration is in a harmonic form for simplicity, its formula can be written as Equation (5), where *ω*_*n*_ = $\sqrt{K/M}$ is the natural angular frequency, and *ω* is excitation frequency. When $\tilde{\omega }=\omega /\omega_{n}$, $\delta =1/{(\omega }_{n}RC_{P})$ and $\gamma =\theta^{2}/\left(KC_{P}\right)$, the output voltage of PB can be calculated as Equation (6), where $\varphi_{v}$ is the phase difference:

(5) $$\ddot{Z}=\omega_{n}^{2}Acos\left(\omega t\right),$$

(6) $$v=\frac{C_{P}V}{\theta }\frac{A\tilde{\omega }}{\sqrt{\Gamma \left({\tilde{\omega }}^{2}+\delta^{2}\right)}}\cos\left(\omega t+\varphi_{v}\right).$$

(7) $$\Gamma =\left({\tilde{\omega }}^{2}-1-\frac{\gamma^{2}}{1+{\left(\delta /\tilde{\omega }\right)}^{2}}\right)+\left(\frac{c\tilde{\omega }}{M\omega_{n}}+\frac{{\delta \gamma }^{2}}{\tilde{\omega }(1+{\left(\delta /\tilde{\omega }\right)}^{2})}\right)$$

**Figure 6:**
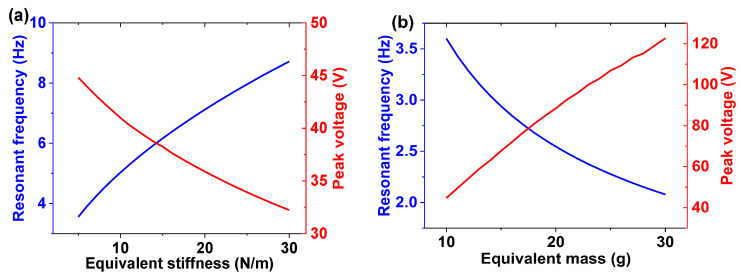
The resonant frequency and peak voltage varied with (**a**) equivalent stiffness (M = 10 g) and (**b**) equivalent mass (K = 5.116 N/m).

In order to initiate the parametric analysis about the features of piezoelectric cantilever beam, a numerical simulation was conducted with the system parameters determined from experimental results. This work was accomplished by testing the piezoelectric cantilever beam using a model identification procedure [37,38]. The identified parameters are listed in Table 1. In the numerical simulation, a 0.2 g sinusoidal acceleration was loaded to excite the cantilever. Figure 6 shows the influences from equivalent mass and stiffness on the device resonant frequency and peak voltage. The equivalent mass can promote the generated peak voltage and lower the resonant frequency. However, a large mass may produce overlarge stress in the beam, which may reduce the system life and even directly damage it. The equivalent stiffness reveals the opposite effect, and its adjustment also needs to be in accord with the target frequency and requirement of long-term stability. Concerning the frequency of daily vibrations, the dimension of PB and the weight of mass in device prototype were determined.

## 3. Experiments and Results

### 3.1. Prototype and Experimental Setup

**Figure 7:**
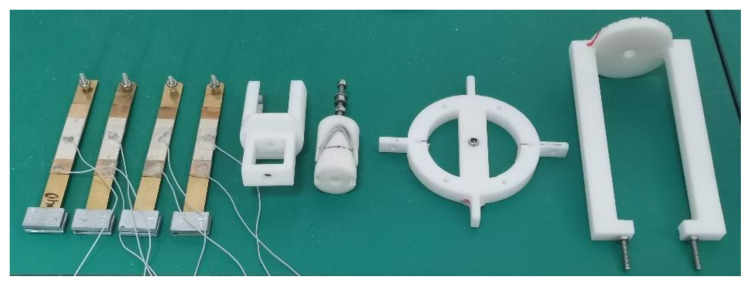
The fabricated components of PVEH prototype.

To examine the feasibility of utilizing the proposed cam rotor in a tri-directional PVEH, a corresponding prototype was fabricated (Figure 7). The turnplate, bracket, cam rotor, and driving fork were 3D printed from UV curable resin (Grade No.: 8111X, Greatsimple Technology, Zhongshan, China) according to the models established in the CAD software (SolidWorks 2018, Dassault Systemes, Waltham, MA, USA). The stereo lithography appearance technique was used to manufacture the components layer by layer. The used parasolid models can be found in Supplementary Materials. The turnplate provided a mounting radius of 42.5 mm to the PBs, and the cam rotor had a radius of 11 mm with a 4 mm deep groove. The transfer bar was made from a 50 mm long M3 thread rod. Each piezoelectric beam was made of a brass substrate (Grade No.: H68), a 10 g iron mass and a piezoelectric patch (Grade No.: PZT-5H, Konghong New Material Corp., Xi’an, China). The brass substrates were the same size of 100 × 10 × 0.2 mm^3^, and the dimension of the PZT patches was 30 × 10 × 0.2 mm^3^. Finally, the PVEH prototype was achieved by assembling these components together with the help of locknuts and bearings (Mode: MF63ZZ, NSK, Tokyo, Japan).

**Figure 8:**
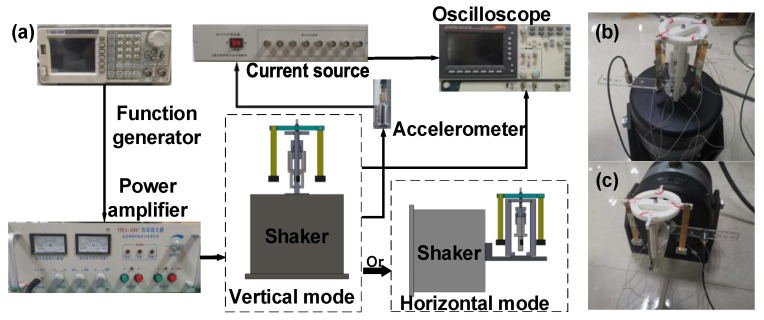
Experimental setup: (**a**) setup composition, (**b**) mounting for vertical vibration and (**c**) mounting for horizontal vibration.

The experimental platform is shown in Figure 8. A sinusoidal signal was generated using the function generator (SDG1020, Siglent, Shenzhen, China) and amplified using the power amplifier (HEA-200C, Funeng, Nanjing, China) to excite the shaker (HEV-200, Funeng, Nanjing, China). An oscilloscope (GDS-1072B, GWINSTEK, Suzhou, China) was used to measure the output voltage of the PVEH prototype and the acceleration signal from the accelerometer (SD14N14, BDHSD, Qinhuangdao, China) through the constant current source (SD14T03, BDHSD, Qinhuangdao, China). With the help of a tailored fixture, the prototype could be stimulated by vertical (Figure 8b) and horizontal (Figure 8c) vibrations.

### 3.2. Characterization Results

**Figure 9:**
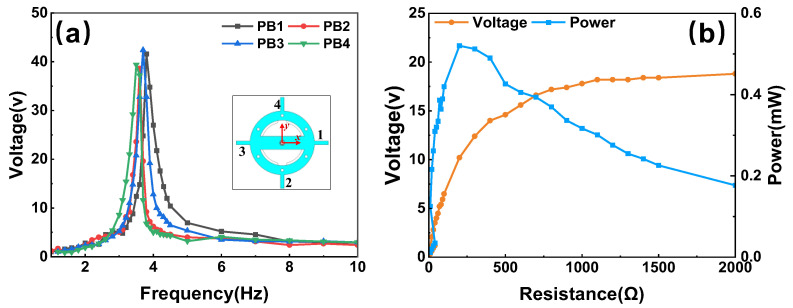
The electrical characterization of PVEH prototype under vertical vibration: (**a**) open-circuit voltage versus frequency and (**b**) optimal impedance and output power.

Firstly, the features of the PVEH prototype in harvesting vertical vibration were characterized. A harmonic acceleration with an amplitude of 0.2 g (1 g = 9.8 m/s^2^) and swept frequency was loaded onto the prototype. The curves of the open-circuit voltages versus the excited frequency are shown in Figure 9a. The tested beams were numbered per the inset. Due to the slight deviation in the production of the brass substrate and PZT adhesion, the output open-circuit voltages and resonant frequencies of the four PBs show little inconformity. The peaks all appear near the value of 3.7 Hz in frequency and 40 V in voltage, and the maximum one of 42.4 V was obtained from No. 3 PB at 3.7 Hz. The optimal impedance and maximum output power of the proposed PVEH under the vertical mode were also tested. The output of the PBs was rectified using a diode bridge and connected in parallel in the experiment. The obtained results under a 3.7 Hz and 0.2 g excitation are shown in Figure 9b. The optimal load resistance is measured as 200 kΩ with a maximum power of 0.52 mW. Then, the power density (maximum power/total volume of prototype) under the 0.2 g vertical vibration is 5749.3 mW/m^3^.

**Figure 10:**
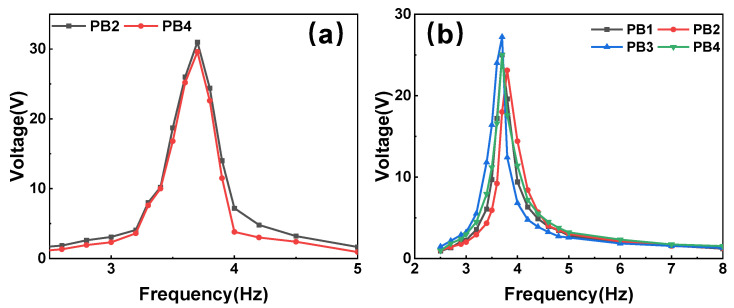
Open-circuit voltage versus frequency under horizontal excitation with different orientations: (**a**) along *x*-axis and (**b**) 45° with *x*-axis.

**Figure 11:**
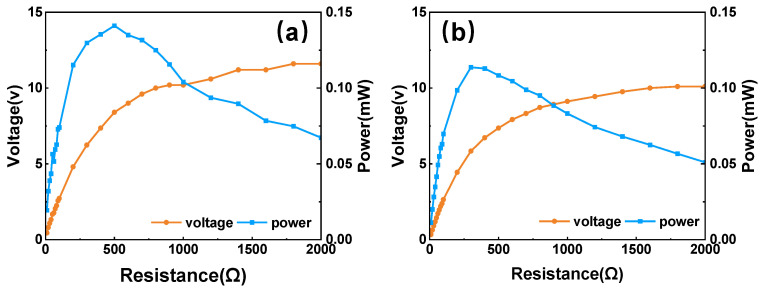
Optimal impedance and output power under horizontal excitation with different orientations: (**a**) along *x*-axis and (**b**) 45° with *x*-axis.

Then, the PVEH features under the horizontal mode were investigated using a similar procedure. Two operation circumstances with different excitation orientations were considered. In the former circumstance, the vibration was loaded along one of the horizontal coordinate axes (*x*-axis in the experiment). The voltage–frequency curves of two triggered PBs are shown in Figure 10a. As the working direction of the other two beams is perpendicular to the excitation direction, nearly no voltage is generated by them. When an angle of 45° is set between the excitation direction and *x*-axis, all PBs are stimulated, and the obtained voltage–frequency curves are shown in Figure 10b. The four PBs still resonate at a frequency around 3.6 Hz with a maximum output voltage of 27.2 V. The output power under the horizontal mode is shown in Figure 11. The rectification and parallel connection were also conducted. When the vibration is along *x*-axis, the maximum power that can be obtained is 0.14 mW at the optimal resistance of 400 kΩ. When the angle turns to 45°, the values change to 0.11 mW and 300 kΩ. These experimental results indicate that the newly proposed cam-rotor-based PVEH has a good performance in harvesting multidirectional vibrations. Then, the power density under the 0.2 g horizontal vibration is 1547.8 mW/m^3^.

### 3.3. Discussions

A simple comparison was conducted between the proposed PVEH and several previously reported multidirectional PVEHs, and the results are summarized in Table 2. The deviation in the last column indicates the performance inconsistency of the referred PVEHs among different axes, which is calculated as

(8) $$Deviation=\max\left(\frac{V_{max}-V_{min}}{V_{max}},\frac{f_{max}-f_{min}}{f_{max}}\right)\times 100\%.$$

*V_max_* and *f_max_* are the maximum values of the measured voltage and frequency; and *V_min_* and *f_min_* are the minimum ones. Among the numerous PVEHs, the cam-rotor-based PVEH in this paper shows considerable consistency in its harvesting performance when treating multidimension vibrations. The maximum deviation of the proposed PVEH originates from the peak voltages under vertical and horizontal conditions. The vertical vibration is first regulated into a circular motion by the cam rotor, and the induced centrifugal acceleration is larger than the vibration amplitude in the proposed prototype. In the meantime, the resonant frequencies at different axes are all determined by the PBs, and the value stabilizes at around 3.7 Hz for both the vertical and horizontal vibrations.

Considering the measured voltage–frequency and power–load curves, some issues require a further explanation. When triggered by the horizontal vibration, the output voltage of each PB varied with the loading direction. Vibration along the bending direction of the cantilever can produce an effective stress in the PZT patch. Only two PBs output favorable voltage when the vibration is along the *x*-axis, and no voltage was generated by the other two orthogonal PBs. When an angle smaller than 90° exists between the vibration and horizontal axis, all PBs can sense a vibration component along its bending direction and then produce a voltage corresponding to the component amplitude. Obviously, the component amplitude is affected by the load direction, and the participation level of each PB varies with the vibration orientation.

Energy harvesting also requires outstanding durability to guarantee service life. Durability mainly contains two aspects: long-term operational reliability and environmental adaptability. In our prototype characterization, tens of hours of tests were conducted, and there was no significant change in the obtained frequency and voltage parameters. It is reported that the service life of piezoelectric harvesting devices can be up to 5–10 years, which is sufficient for many self-sustaining monitoring applications [39,40]. As for environmental adaptability, temperature is an often-referred parameter. It has been reported that the PZT patch cannot achieve long-term stability when the temperature is above 170 °C due to gradual thermal depolarization, and it will lose its piezoelectric property when the temperature is above its Curie point (about 350 °C for PZT-5H). In the meantime, the resin for the 3D printed prototype components will partially lose its mechanical strength when the temperature is higher than 65 °C. Therefore, the manufactured laboratorial prototype cannot operate at a temperature above 65 °C, but the upper limit can be promoted to about 170 °C if the component material is improved.

### 3.4. Applications

**Figure 12:**
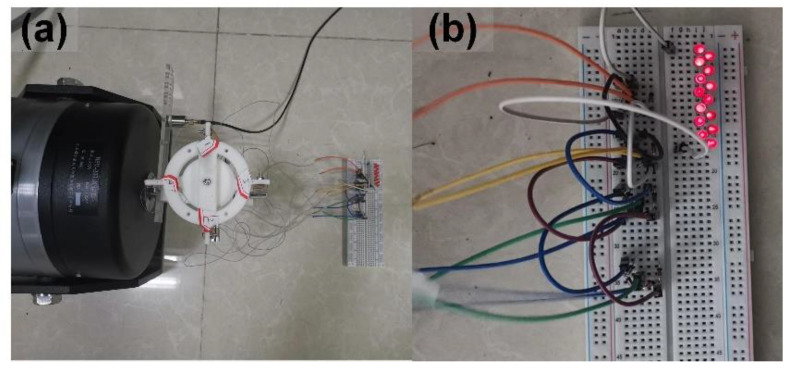
Fourteen LEDs lit up by the PVEH prototype: (**a**) photograph of devices and (**b**) photograph of lighted LEDs.

This subsection validates the capacity of the PVEH prototype in powering low-consumption devices, demonstrating its potential as a practical power source. The application of using the PVEH to light up an LED array is first verified. After the necessary rectification, the parallel connected PBs can light up 14 LEDs when the prototype is triggered by a horizontal 0.2 g and 3.6 Hz vibration (Figure 12).

**Figure 13:**
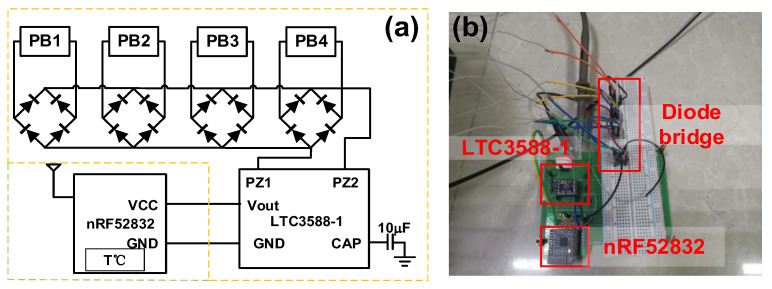
The realized self-sustained WSN system: (**a**) circuit diagram and (**b**) photograph.

**Figure 14:**
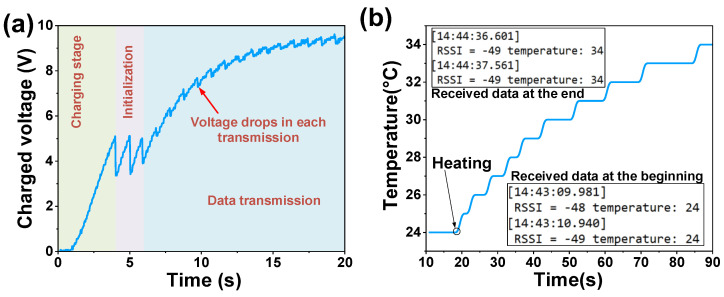
Assessment of PVEH-based WSN system: (**a**) the voltage over energy storage capacitor at different stages and (**b**) the temperature results received by computer.

The output power from a PVEH is usually unstable due to variations in the vibration parameters, so an energy manager is needed to achieve a more stable power source. Herein, we proposed a simple energy management circuit for demonstration, with the purpose of realizing a self-sustained WSN system (Figure 13). The autonomous WSN consisted of a PVEH prototype, four-diode bridge for rectification, an LTC3588-1-based power management circuit for DC-DC conversion with a 10 μF capacitor for energy storage and an nRF52832 board as a Bluetooth transceiver. The sensing data were obtained from a temperature sensor in the Bluetooth board. The voltage over the 10 μF capacitor during data transmission is shown in Figure 14a. The capacitor could be charged to 5.05 V, the under voltage lockout threshold of the LTC3588-1, within 2 s when the PVEH was excited by a vertical 0.2 g and 3.6 Hz vibration. Then, the Bluetooth transceiver was activated and initialized for operation, which caused an obvious decrease in the capacitor voltage. After transceiver regulation, power was stably supplied to the WSN, and the measured temperature was transmitted to the PC terminal through the Bluetooth network. The charged voltage slightly dropped in every transmission. The received temperature data under a heating condition are shown in Figure 14b, and the insets are the screenshots of the data received by the computer.

## 4. Conclusions

This paper reports a cam-rotor-based piezoelectric vibration energy harvester for low-frequency, multidirectional vibrations in daily environments. With the superiority of the cam rotor mechanism in converting vertical linear motion into reciprocating circular motion, the proposed device can use the same piezoelectric beams to harvest energy from multidirectional vibrations with a similar resonant frequency and output voltage. The peak output voltages under different excitation orientations all appear at a frequency around 3.7 Hz. The measured maximum voltage under a 0.2 g vertical vibration is 42.4 V with a power of 0.52 mW. The participation level of each piezoelectric beam varies with the vibration orientation at the horizontal stage, and the obtained energy can simultaneously light up 14 LEDs. A wireless sensing network is sustained based on the output energy of the harvester prototype, and the measured temperature information is successfully transmitted to the receiver. This research exhibits the promising application of a cam rotor in constructing a multidirectional PVEH as well as the potential of applying this PVEH as a power source for low-consumption engineering devices. Further research on optimizing the geometry of the groove in the cam rotor and the hump in the driving fork is still needed to increase the motion conversion efficiency. The next step also includes designing specialized interface circuits to realize high-efficiency energy management.

## Figures and Tables

**Table 1:** Identified system parameters in the equivalent lumped-parameter mode.

Parameter	K(N/m)	M (g)	*Θ* (mN/V)	Cp (nF)	c (N∙s/m)	R (kΩ)
Value	5.116	10	0.062	53	0.01	400

**Table 2:** Comparison of the proposed PVEH with the existing PVEHs.

References	Material	Acc. (g)	Voltage (V)			Frequency (Hz)			Deviation
			*x*	*y*	*z*	*x*	*y*	*z*	
[23]	PZT-5H	0.5	12	32.8	13.6	9	8	6	63.4%
[24]	PVDF	0.5	2.18	16.48	0.685	22.37	22.37	41.47	95.8%
[26]	PZT-5H	0.008	3.01	3.01	3.29	4.56	4.56	8.98	49.2%
[29]	PZT-5H	1.08	11.32	22.55	26.25	4.99	4.99	4.99	57%
[34]	PZT-5A	1.0	13	13	6.4	2.6	2.6	2.9	50.8%
[35]	PZT-5A	1.0	35	12.2	56	2.5	20.4	11.4	87.7%
This work	PZT-5H	0.2	31.2	31.2	42.4	3.7	3.7	3.6	26.4%

## Data Availability

The data presented in this study are available upon request from the corresponding author. The data are not publicly available due to the intellectual property.

## Supplementary Materials

The following supporting information can be downloaded at: https://www.mdpi.com/article/10.3390/mi14061159/s1, Zip File: the parasolid models for the 3D printed components.
